# Intrauterine blood transfusion causes dose- and time-dependent signal alterations in the liver and the spleen on fetal magnetic resonance imaging

**DOI:** 10.1007/s00330-024-11228-y

**Published:** 2024-12-11

**Authors:** Michael Schwarz, Victor Ulrich Schmidbauer, Jakob Malik, Nikolaus Michael Nowak, Patric Kienast, Martin Watzenboeck, Marlene Stuempflen, Caroline Schwarz, Jakob Kittinger, Dieter Bettelheim, Christina Haberl, Julia Binder, Herbert Kiss, Thomas Reiberger, Daniela Prayer, Gregor Kasprian

**Affiliations:** 1 Division of Gastroenterology and Hepatology, Department of Medicine III, Medical University of Vienna, Vienna, Austria; 2 Department of Biomedical Imaging and Image-guided Therapy, Medical University of Vienna, Vienna, Austria; 3 Department of Obstetrics and Gynecology, Division of Obstetrics and Feto-Maternal Medicine, Medical University of Vienna, Vienna, Austria

**Keywords:** Fetal MRI, Intrauterine transfusions, Iron overload, Hepatosplenomegaly, Liver

## Abstract

**Background:**

Intrauterine transfusions (IUTs) are a life-saving treatment for fetal anemia. However, with each transfusion, iron bypasses uptake regulation through the placenta and accumulates in fetal organs. Unlike other imaging modalities, fetal magnetic resonance imaging (MRI) is capable of non-invasively assessing fetal liver disease and/or organ iron overload. This study aimed to investigate the effects of IUTs on MRI findings in the fetal liver and spleen.

**Study design:**

For this retrospective study, we included eight fetuses undergoing IUT and prenatal MRI from 2014 to 2023. The fetuses were gestational age-matched with a cohort that received fetal MRI for other indications, but no IUTs. Signal intensity (SI) and volumetric analyses of the liver and the spleen were performed.

**Results:**

Fetuses receiving transfusions had significantly larger volumes of both liver (*p* = 0.003) and spleen (*p* = 0.029). T1 SI inversely correlated with the number of IUTs (Pearson’s *r* = −0.43, *p* = 0.099). This effect regressed over time (*r* = 0.69, *p* = 0.057). T2 SI did not correlate significantly with transfusion frequency but showed a strong positive correlation with the number of days between IUT and MRI (*r* = 0.91, *p* = 0.002). For splenic SI measures, similar effects were observed regarding T1 SI reduction per received transfusion (*r* = −0.36, *p* = 0.167) and recovery of T2 SI after IUT (*r* = 0.88, *p* = 0.004).

**Conclusion:**

This is the first study to report the effects of IUTs on MRI data of fetal livers and spleens. We observed considerable dose- and time-dependent SI alterations of the liver and spleen following IUT. Furthermore, fetal hepatosplenomegaly can be expected following IUT.

**Key Points:**

***Question*** *What fetal changes are found by MRI after life-saving intrauterine transfusion (IUT)?*

***Findings*** *Dose- and time-dependent reductions in signal intensity of the fetal liver and spleen, as well as hepatosplenomegaly, were found after intrauterine transfusion.*

***Clinical relevance*** *Intrauterine transfusions cause transient iron overload with consequential changes in MRI signal intensity of fetal livers and spleens. Fetal hepatosplenomegaly can be expected following transfusions. Radiologists’ awareness of changes following IUT may improve report quality*.

**Graphical Abstract:**

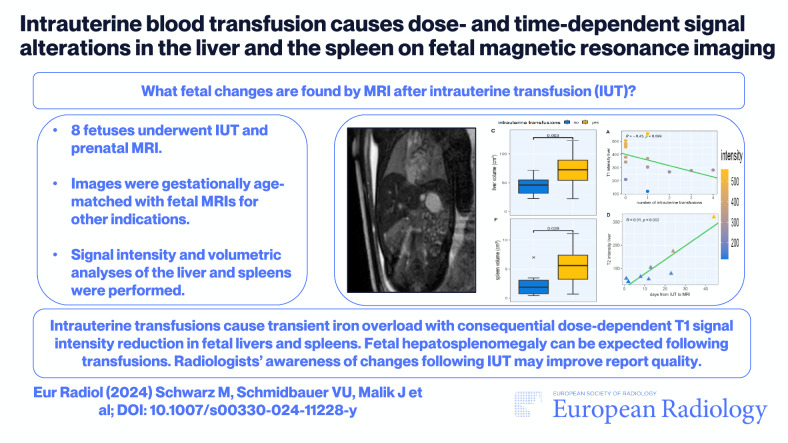

## Introduction

Intrauterine transfusions (IUTs) are a life-saving intervention for severe fetal anemia and, since their introduction in the 1960s, have revolutionized the management of this potentially lethal pregnancy complication [1, 2]. The most common indication for IUT is hemolytic disease of the fetus or newborn (HDFN), an incompatibility of maternal and fetal AB0 blood types or rhesus factor that results in life-threatening fetal hemolysis and anemia [3]. Other causes of fetal anemia that may require IUT are parvovirus B19 infections, feto-maternal hemorrhage, twin-to-twin transfusion syndrome, or placental tumors [3, 4]. While IUT has proven to be an invaluable tool, the effects of IUTs and the resultant iron exposure on fetal organs are yet to be evaluated.

As the human body has no direct method by which to excrete excess iron, it accumulates in organs such as the liver, the heart, or the pancreas, and, through oxidative stress, causes cell damage and organ dysfunction [5, 6]. During pregnancy, fetal iron uptake is strictly regulated through the placenta, which ensures a balance between a sufficient supply for fetal development and the prevention of iron overload with potential organ damage [7, 8]. However, during an IUT, donor blood—and, therefore, iron—is transfused directly into the fetal bloodstream at the placental or intrahepatic umbilical vein, thus bypassing the placenta [9]. Repeated transfusions, which anemic fetuses often require, can lead to hepatic iron overload [2, 10–12], sometimes even resulting in post-natal hepatitis [13].

The liver is the major receiving organ of excess iron, where it is stored as ferritin or hemosiderin [6]. On magnetic resonance imaging (MRI), the presence of ferritin in tissue initially shortens the relaxation times and may cause darkening effects of the affected organs on various sequence types [14, 15]. In children, adults, and in utero, MRI can be used to assess the hepatic iron load [16–18]. However, imaging studies usually focus on adult subjects and the long-term effects of iron overload, while data regarding the immediate impact of IUTs on fetal organs are lacking. Herein, we present the first study to analyze the effect of IUTs on prenatal MRI findings.

## Methods

### Subjects and ethics

We retrospectively included women undergoing both IUTs and subsequent fetal MRI at a tertiary care center in Vienna, Austria, from July 2014 through May 2023. Written informed consent was obtained before IUT and fetal MRI. Maternal medical history, indication for IUT, and gestational age at the time of MRI (weeks + days) were assessed at baseline. For statistical analysis, the cohort was matched by gestational age in a 1:1 ratio with other fetuses receiving fetal MRI without IUT and for other indications. We excluded fetuses with apparent liver defects. This study was conducted in accordance with the principles of the Declaration of Helsinki, as approved by the local ethics committee.

### Fetal MRI

Imaging was performed according to the guidelines for fetal MRI, as proposed by the International Society of Ultrasound in Obstetrics and Gynecology [19]. All fetuses were imaged based on a standardized fetal MR protocol (Suppl. Table S1) on one Philips Ingenia 1.5 Tesla MR system equipped with a body coil. Imaging included T1- and T2-weighted sequences in three orthogonal planes of the fetal upper abdomen. Fetal T1- and T2-based signal intensity (SI) measurements were obtained directly on a Picture Archiving and Communication System (PACS) workstation. Volumetric analyses of the fetal liver and spleen were performed using ITK-SNAP 4.0.1 (Penn Image Computing and Science Laboratory (PICSL), University of Pennsylvania) on T2-weighted imaging data. Assessment of SIs and organ volumes was conducted by two doctors independently and blinded.

### Statistics

Calculations and visualization were performed in RStudio build 386 (Posit Software PBC). Variables are presented as absolute numbers (and percent) and mean (and standard deviation). The normal distribution of variables was assessed using the Kolmogorov–Smirnov test. Two-sided Student’s paired *t*-tests were used to compare the SI measures and volumetric data between the two groups. Pearson’s correlation coefficient (Pearson’s *r*) was used to detect (a) relationships between the number of IUTs and SI alterations; and (b) relationships between the timespan after IUT and SI alterations. The level of significance was set to a *p*-value of 0.05. Interrater reliability was assessed utilizing the intraclass correlation coefficient (ICC), a two-way mixed-effects model for absolute agreement. ICC results were interpreted as proposed by Koo and Li, with values of 0.90 or greater indicating “excellent agreement” [20].

## Results

### Patient and transfusion details

In total, our cohort consisted of eight patients undergoing IUT and fetal MRI and eight gestational age-matched controls who did not receive IUTs, but fetal MRI for other indications. At the time of the MRI, the mean maternal age was 30.8 years (SD 5.8) for the IUT group and 31.3 years (SD 4.7) for the control group. The mean gestational age was 27.1 weeks (SD 2.9) for IUT and 27.2 (SD 3.1) for controls. The maximum difference in gestational age between the matched pairs was six days. In the IUT cohort, the mean number of total IUTs received per fetus was 3.4 (SD 2.2). However, some patients underwent fetal MRI during ongoing transfusion sessions. Every patient received one to four IUTs before imaging of the liver (mean IUTs per patient before MRI: 1.9 (SD 1.1)). Of our eight included patients, five (62.5%) received IUTs for hemolytic disease of the fetus and newborn (HDFN), two (25.0%) for intrauterine parvovirus B19 infection, and one (12.5%) for twin-to-twin transfusion syndrome. Per session, 51 mL (SD 21 mL) were transfused on average. See Table 1. Detailed information regarding the individual IUT sessions is provided in Suppl. Table S2.

**Table 1:** Characteristics of the IUT cohort and gestational age-matched controls

Pat	Mat. age	Gest. age	Indication	IUTs	Days	Con	Mat. age	Gest. age	Indication
1	29	22 + 2	HDFN	3/7	1	A	36	22 + 1	kidney cyst, duod. atr.
2	35	24 + 4	Parvovirus	1/1	23	B	25	25 + 1	cleft palate
3	36	25 + 6	HDFN	2/4	13	C	29	25 + 3	kidney agenesis
4	33	26 + 0	HDFN	2/5	8	D	37	26 + 0	distended bowel loop
5	31	28 + 5	HDFN	1/2	24	E	30	28 + 4	polyhydramnion
6	18	28 + 6	TTTS	1/1	44	F	30	28 + 4	liver alterations on US
7	35	29 + 4	HDFN	1/1	12	G	25	29 + 5	CCAM cleft
8	29	31 + 1	Parvovirus	4/4	2	H	34	32 + 0	mega cisterna magna

Gestational and maternal age were calculated for the date of imaging. The main reason for IUT was HDFN (five of eight patients). Others were infections with parvovirus B19 (two patients) and TTTS (one patient). Some patients had their MRI during still ongoing IUT sessions. Listed under “IUTs” are the number of IUTs performed before MRI, followed by the total number of IUTs received (before/total). The column “days” shows the number of days between the last IUT and fetal MRI. In the gestational age-matched control cohort, no transfusions were performed, and there were no cases of fetal liver disease. Indications for MRI in the control cohort are listed on the right. The patient with nonspecific liver parenchyma on ultrasound had no correlating pathology on MRI

*con* control, *CCAM* congenital cystic adenomatoid malformation of the lung, *duod. atr.* duodenal atresia, *gest. age* gestational age, *HDFN* hemolytic disease of the fetus and newborn, *IUT* intrauterine transfusions, *mat. age* maternal age, *pat* patient, *US* ultrasound

### Interrater reliability

We found an excellent level of agreement between both observers regarding the assessment of T1 SI of the liver (ICC 0.985, 95% confidence interval (CI) 0.954–0.995) and spleen (ICC 0.967, 95% CI 0.961–9.996) and T2 SI of the liver (ICC 0.987, 95% CI 0.961–0.996) and spleen (ICC 0.970, 95% CI 0.907–0.989). For organ volume assessment, we found excellent reliability for volume assessment of the liver (ICC 0.972, 95% CI 0.322–0.994) and spleen (ICC 0.903, 95% CI 0.181–0.976).

### Radiographic changes of livers and spleens following IUT

**Fig. 1:**
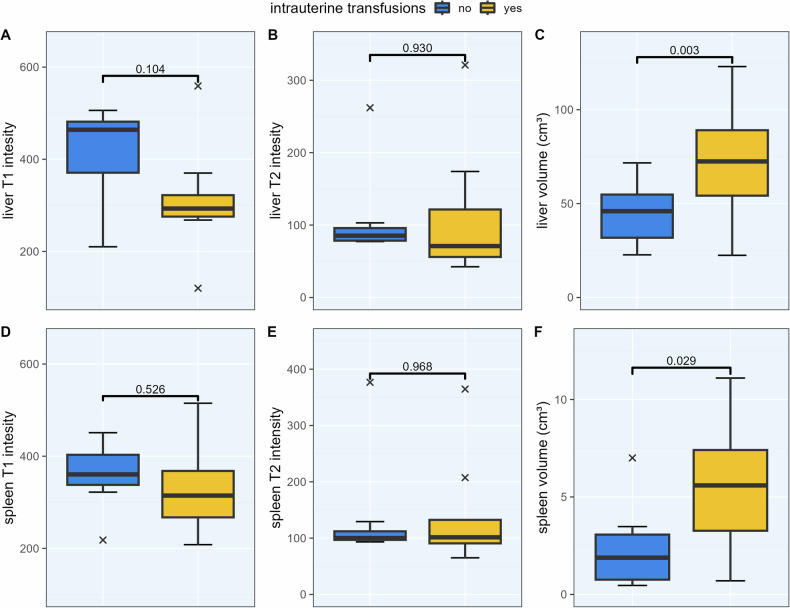
T1 and T2 signal intensities, as well as organ volumes of fetal liver and spleen. Fetuses who received intrauterine transfusions (IUTs) exhibited a numerical reduction in T1-weighted signal intensity of the liver and spleen compared to controls (panel **A** & **D**). This reduction was not significant when not adjusted for time or number of transfusions. This trend was less pronounced for T2-weighted imaging (panel **B** & **E**). Fetal hepatosplenomegaly was commonly found following IUTs shown here by significantly increased volumes of fetal liver and spleen (panel **C** & **F**). Group comparison via paired *t*-test

Compared to gestational age-matched controls, fetuses receiving IUTs had numerically lower SI on T1-weighted imaging data for both fetal livers and spleens, although not significantly (*p* = 0.104 and *p* = 0.526, respectively). No significant differences in SI measures were found on T2-weighted imaging between the groups for liver (*p* = 0.930) or spleen SI (0.968). However, fetuses who received IUT had significantly larger volumes of both liver (*p* = 0.003) and spleen (*p* = 0.029), when compared to the control group. For details, see Table 2. Data are visualized in Fig. 1.

**Table 2:** Comparison of T1 and T2 signal intensities (SI) as well as organ volume between fetuses who received IUTs and those who did not

Imaging findings	No IUT	IUT	*p*-value
Mean signal intensity (standard deviation)			
Liver T1	416 (100.0)	311 (123.0)	0.104
Liver T2	108 (62.9)	112 (94.4)	0.930
Spleen T1	359 (71.5)	328 (103.0)	0.526
Spleen T2	137 (97.4)	140 (101.0)	0.968
Mean organ volumes in cm³ (SD)			
Liver volume	45.1 (17.2)	71.9 (32.5)	**0.003**
Spleen volume	2.4 (2.2)	5.5 (3.7)	**0.029**

Fetuses who received IUTs had larger volumes of the liver and spleen. IUTs led to a numerical reduction in T1-weighted SI, although not statistically significant. Results are visualized in Fig. 3. Group comparison via paired *t*-test, *p*-values < 0.05 shown in bold.

**Fig. 2:**
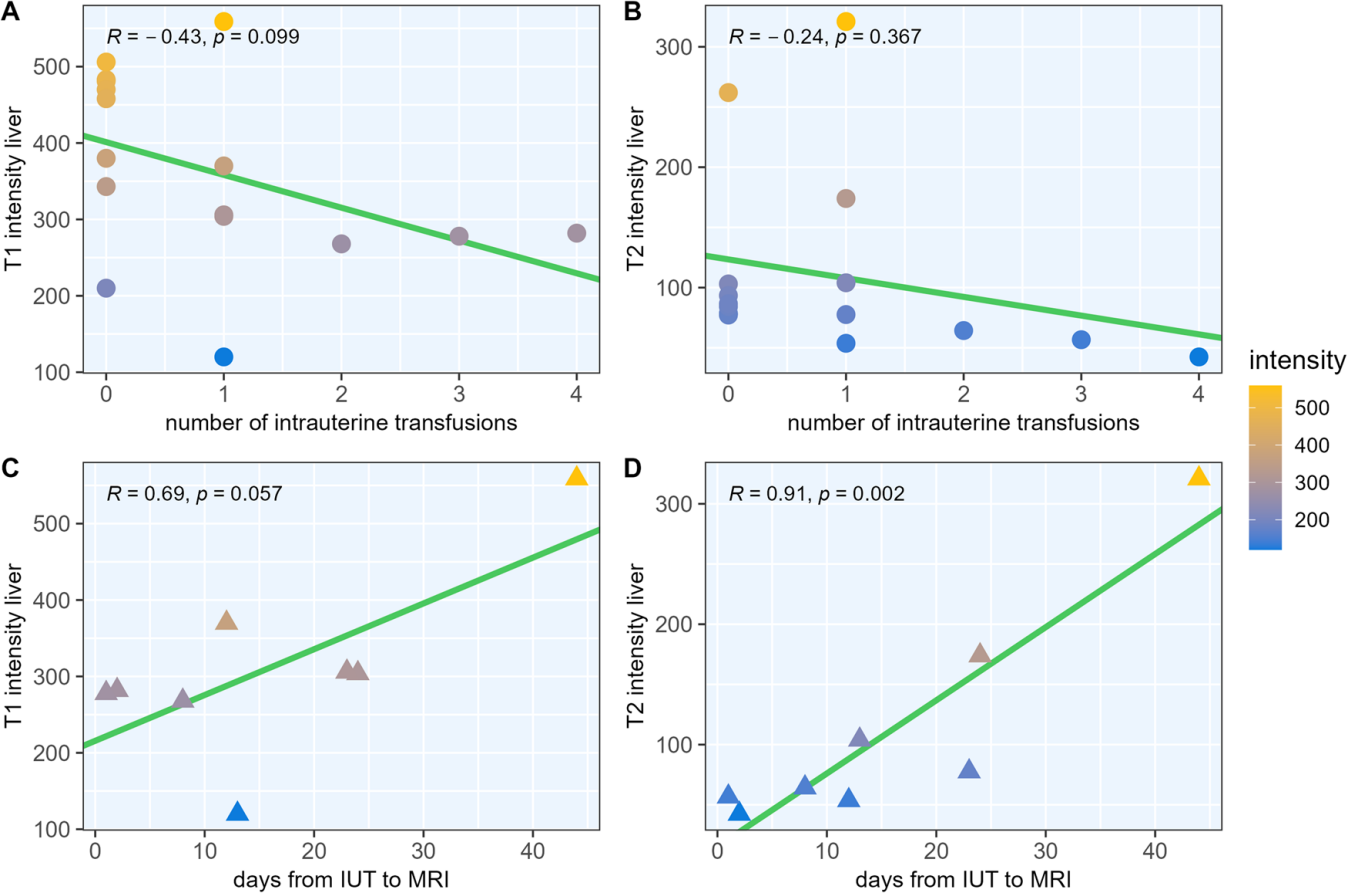
Fetal liver T1 and T2 signal intensities by the number of transfusions (top row) and number of days after intrauterine transfusion (IUT; bottom row). On T1-weighted imaging, the signal intensity of the liver decreased with the number of transfusions received and then increased again with the number of days elapsed since transfusion (**A**, **C**, n.s.). This dose-dependent effect was not seen on T2-weighted imaging as one IUT already led to a pronounced reduction in signal intensity (**B**, n.s.). However, this transfusion-induced reduction in signal intensity also regressed over time (**D**). Note that the top panel (**A**, **B**) also included gestational age-matched controls who did not receive any IUT (circles), while the bottom panel (**C**, **D**), which stratifies data by elapsed days since IUT, only includes fetuses who received IUTs (triangles). Assessment of linear correlation using Pearson’s correlation coefficient

We then stratified our cohort according to the number of IUTs received and to the days elapsed since IUT to account for dose- and time-dependent changes. We found an inverse correlation between T1 SI of the fetal liver and the number of IUTs received (Pearson’s *r* = −0.43, *p* = 0.099). On T2-weighted imaging, already one IUT led to a drastic reduction in SI, and no relevant correlation or trend was observed (*r* = −0.24, *p* = 0.367). When looking at the number of days elapsed between the last IUT and MRI (median number of days 12.5, interquartile range 6.5–23.5 days) on T1-weighted imaging, we found that the reduction in SI recovered with the number of elapsed days, however, not significantly (*r* = 0.69, *p* = 0.057). However, for T2-weighted imaging, we found a marked reduction in SI, most profound in temporal proximity to the IUT that then regressed with elapsed time, translating in a highly significant and strong correlation between SI and elapsed days (*r* = 0.91, *p* = 0.002). For details, see Fig. 2, and for example images, see Suppl. Fig. S1.

**Fig. 3:**
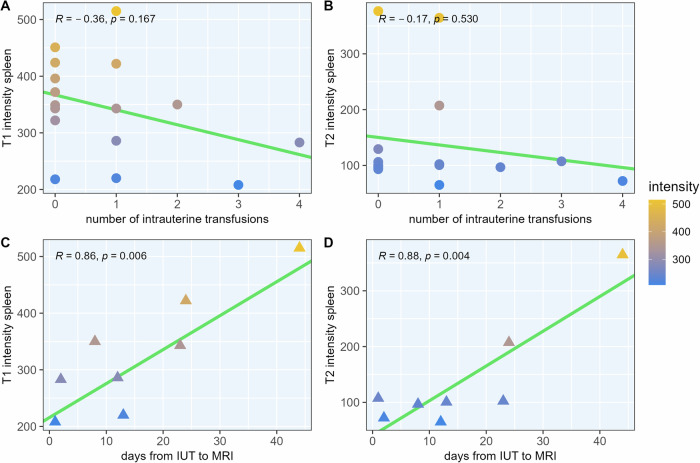
Fetal spleen T1 and T2 signal intensities by the number of transfusions (top row) and number of days after intrauterine transfusion (IUT; bottom row). Compared to the observations in fetal livers, similar effects of IUTs on SI were seen. Again, on T1-weighted data, signal intensity reduced with the number of transfusions (**A**, n.s.), which recovered over time with each day passed since the IUT (**C**). On T2-weighted imaging, no significant correlation was found regarding dose (**B**), however, signal intensity recovered over time (**D**). The top panel again includes controls without IUTs (circles), while the bottom panel only shows fetuses who received IUTs (triangles). Assessment of linear correlation using Pearson’s correlation coefficient

Similar effects were observed for fetal spleen imaging: T1 SI decreased with the number of IUTs (*r* = −0.36, *p* = 0.167) and then recovered with the number of days elapsed (*r* = 0.86, *p* = 0.006). On T2 imaging, again, no significant correlation was found with the number of IUTs (*r* = −0.17, *p* = 0.530), but the reduced SI recovered over time in a significant manner (*r* = 0.88, *p* = 0.004). For details, see Fig. 3 and Suppl. Fig. S2.

### Fetal outcomes

Regarding fetal outcomes, we observed only one intrauterine demise during pregnancy and no fetal deaths during or immediately after birth. Seven children were born by C-section and one by spontaneous birth. We observed prolonged jaundice after birth in three newborns, each of whom received four or more IUTs before delivery. One of these newborns had steatosis and hepatosplenomegaly on ultrasound two weeks after birth, which was self-limiting. No lasting liver or spleen damage was reported for any patient. One newborn experienced intrauterine hypoxia during birth and developed encephalopathy and a developmental handicap. Two newborns experienced another episode of hemolysis post-partum and required transfusions. All except one patient are alive, according to the information provided to our center. No remarkable maternal outcomes were reported during follow-up. For details on post-partum outcomes, please see Suppl. Table S3.

## Discussion

**Fig. 4:**
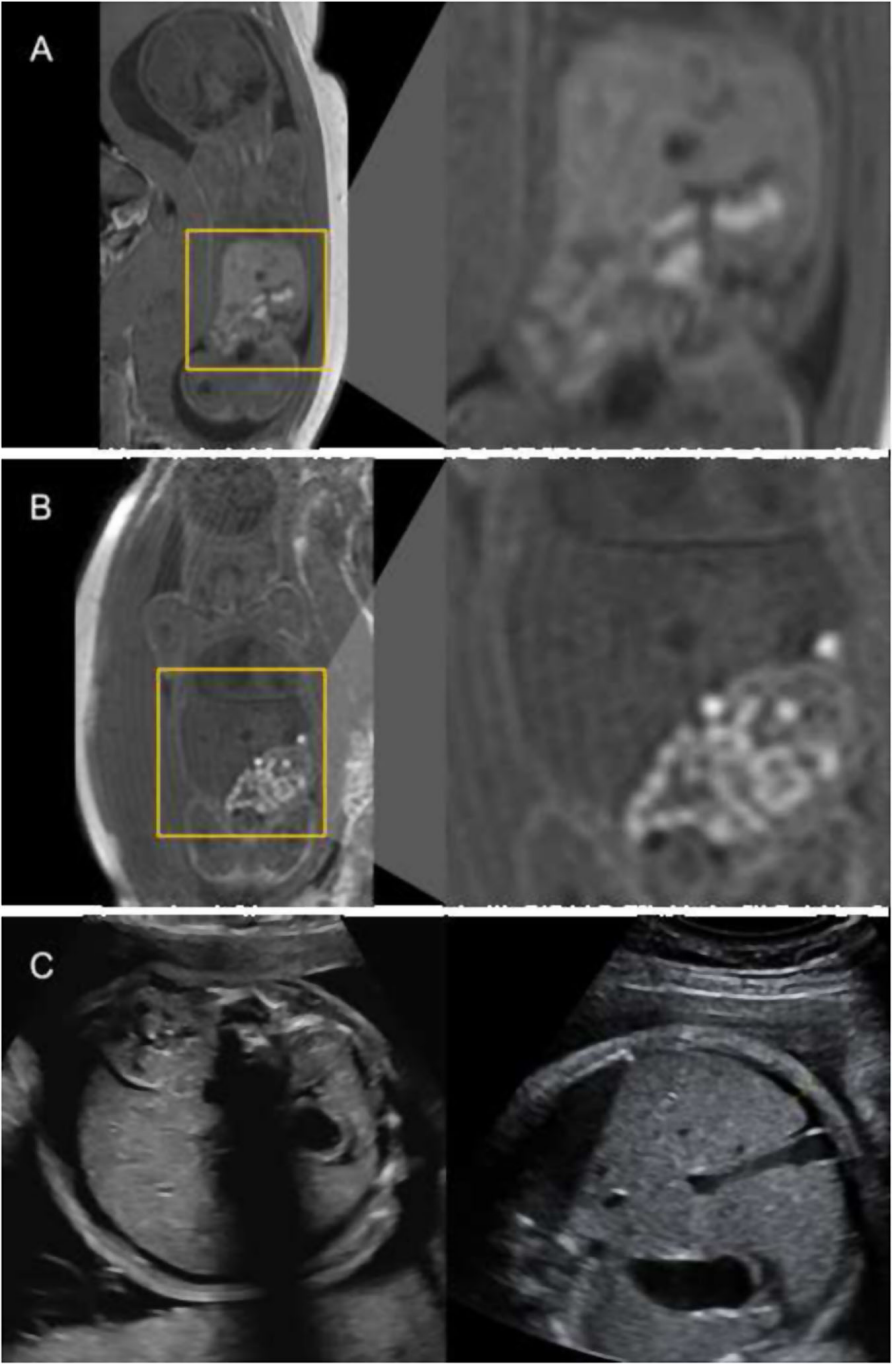
Effects of intrauterine transfusions on fetal liver parenchyma on T1-weighted magnetic resonance (**A**, **B**) and ultrasound imaging (**C**). **A** Picture A shows a fetus at a gestational age of 28 + 4 weeks, who did not receive IUT. On T1-weighted sequences, the liver parenchyma imposes hyperintensely (signal intensity (SI) = 458) compared to surrounding tissues. **B** Images of a different fetus at a gestational age of 28 + 5 weeks, who received a total of two IUTs, the last one 24 days prior to MRI acquisition. Compared to the fetus who did not receive IUT, the liver parenchyma appears darker (SI = 304) on T1-weighted sequences, even though the procedure was performed several weeks prior. **C** For comparison, ultrasound images of a third fetus (examinations from gestational age 29 + 0 and 30 + 0) are shown. The fetus received a total of five IUTs during pregnancy, one between the two ultrasound examinations. No apparent effects on parenchyma echogenicity were detectable. The thin lamella of ascitic fluid was most likely due to the known parvovirus B19 infection

**Fig. 5:**
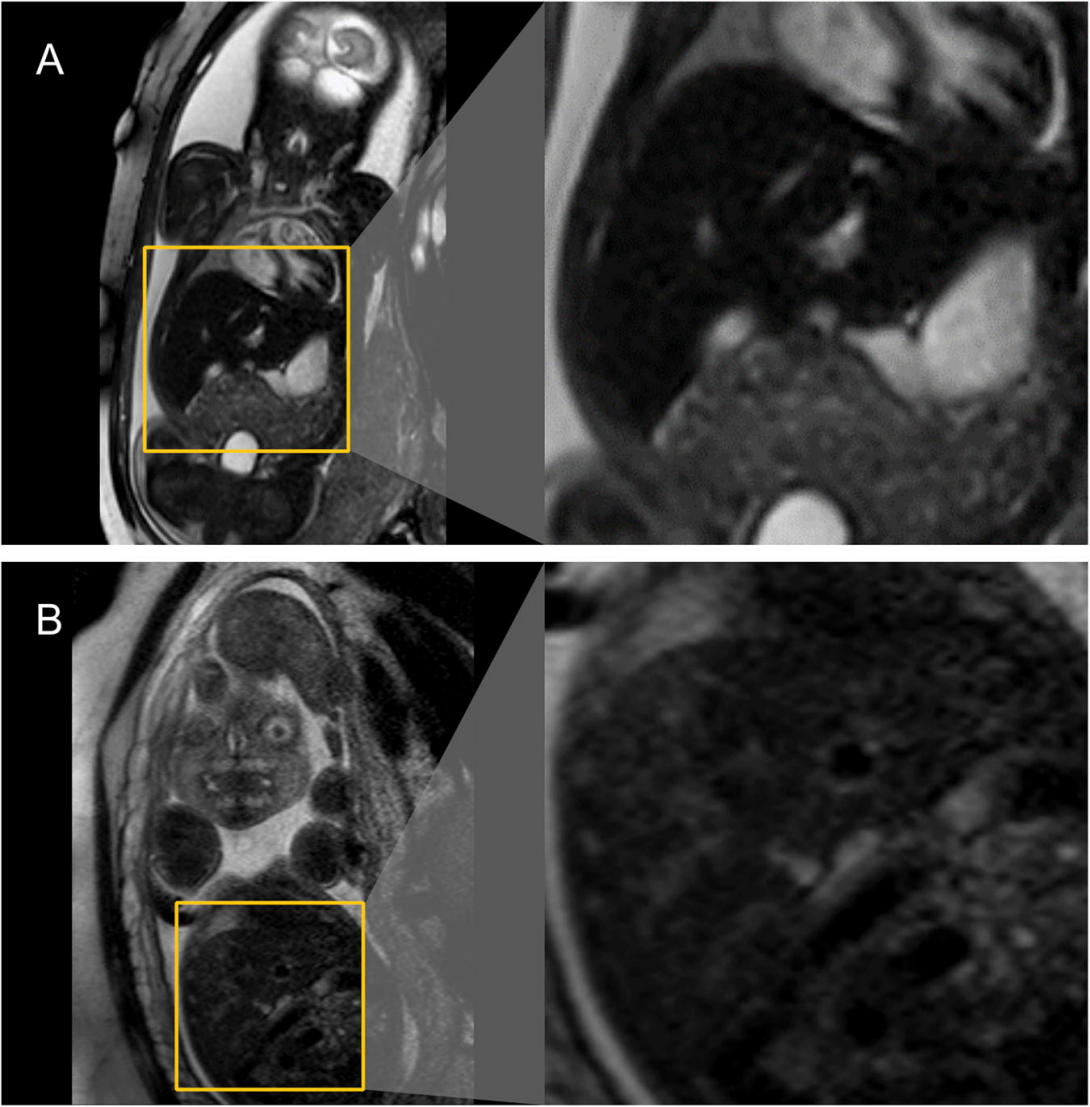
Time-dependent effects of intrauterine transfusions on fetal liver parenchyma on T2-weighted magnetic resonance imaging. **A** T2-weighted MRI sequence of a fetus at a gestational age of 31 + 1 weeks, who received a total of five IUTs, the last one three days prior to MRI acquisition. On T2-weighted sequences, a profound reduction in the signal intensity of the fetal liver parenchyma is seen (SI = 42). **B** Images of a different fetus at a gestational age of 28 + 6 weeks, who received one IUT in total, 44 days prior to MRI acquisition. After this time delay of several weeks between IUT and MRI, the SI of the fetal liver steadily increased and no longer shows the profound signal intensity reduction seen in **A** (SI = 321)

In this study, we investigated the potential of prenatal MRI to visualize iron accumulation in the fetal liver and spleen after IUTs. We were able to detect time- and dose-dependent radiological changes following prenatal fetal blood transfusion. On T1-weighted imaging, we observed decreases in SI of fetal livers and spleens that became more prominent with the number of IUTs received; this effect regressed over time. While both correlations were not statistically significant (*p* = 0.099 and *p* = 0.057), a clear trend was observed, and the data was in itself conclusive. On the other hand, on T2-weighted imaging data, the effect of IUTs on the intensity of the liver and spleen was so pronounced after only one transfusion that no dose-dependent effect could be determined. However, the profound reduction in SI was most pronounced in temporal proximity to the transfusion and showed a highly significant time-dependent regression. So T1-weighted imaging was able to capture dose-dependent and additive changes in SI of the liver and spleen following IUT, while T2-weighted imaging excelled in portraying the time-dependent regression of this effect. Besides changes in SI, fetuses who received IUTs also had significantly larger liver and spleen volumes on imaging. See Figs. 4 and 5 for MRI and ultrasound images; Fig. 1 for statistical analysis.

In animals, children, and adults, excess iron from blood transfusions accumulates in the liver and the spleen [21–23]. Previous studies have shown that fetuses who receive IUTs have highly elevated serum ferritin levels (above the 95th percentile) at birth [24], and the number of transfusions, as well as the amount of blood transfused, correlates with the amount of iron deposits in the liver and spleen found post mortem [10]. However, post-natal ferritin levels slowly decrease as the abundant iron is used for physiological processes over time [24]. In conditions with hereditary iron overload like hemochromatosis, hepatic iron accumulation reduces hepatic SI, while iron depletion through frequent phlebotomies can restore normal signaling [25]. Similar findings have been described in patients with hepatic iron overload due to frequent blood transfusions for hematological disorders such as thalassemia, in which iron chelation therapy was administered, and thus, the hepatic iron load was reduced [18, 26]. However, for our observed cohort, the intrauterine timeframe was presumably too limited for relevant redistribution of excess iron stored in the hepatic and splenic tissue to explain the observed time-dependent regression of T2-weighted SI reduction.

Accumulation of free hepatic iron leads to the formation of reactive oxygen species, which damages cell organelles, cell membranes, and DNA, which, in turn, causes hepatic inflammation, fibrogenesis, and ultimately, liver cirrhosis [5, 6]. On MRI, inflammation of the liver results in increased relaxation times and, thus, SI changes [27]. Regarding the dynamic of T2 signal changes we observed, it could be argued that, after the initial profound reduction in T2 SI following IUT, the subsequent rise in SI can be interpreted as a recovery but also as ongoing inflammation of the liver due to oxidative stress.

While T1-weighted signaling is not the method of choice with which to analyze hepatic iron load (which is usually done in T2/T2* sequences), T1 SI is still affected by the presence of iron in the liver [17, 28]. We observed a dose-dependent effect of T1-weighted SI reduction in correlation with the number of transfusions received by the fetus and argue that this may be an additive effect of accumulating iron atoms in the fetal liver. While SI on T1 is reduced by iron overload, accompanying inflammation appears not to induce considerable T1 SI changes in the liver [28], which might explain the less pronounced time-dependent effect in T1 compared to T2 signaling we observed.

Fetuses who received IUTs had significantly larger livers and spleens compared to controls (see Fig. 1). We think this can be attributed to the relevant volume load for the fetal organism due to IUTs. In severe fetal anemia, it can be necessary to transfuse 10% or more of the fetal body weight in several sessions [4]—for an adult with a body weight of 80 kg, this would translate into eight liters or approximately 25 transfusions. Guidelines on fetal transfusions recommend that the fetal hematocrit should not be elevated more than four-fold from baseline to avoid overwhelming the cardiovascular system [1]. In adults [29] and children [30] alike, typical causes of splenomegaly are infections (such as Epstein-Barr virus or cytomegaly virus), hematologic disorders (such as leukemias or lymphomas, but also diseases with a high turnover of red blood cells such as sickle cell anemia, thalassemia, or spherocytosis), congestion (liver cirrhosis or venous thrombosis), inflammatory diseases (rheumatoid arthritis, lupus, sarcoidosis, etc.), or infiltrative diseases (such as Gaucher’s disease, amyloidosis, etc.). In our patients, congestive splenomegaly was a possible cause for the observed enlargement and splenic rupture following massive IUT has been described before [31].

We observed the MRI appearance of a “transient hemochromatosis” of the liver and spleen as well as a transient hepatosplenomegaly following IUTs. For the radiologist interpreting the described finding, it is important to consider the patient’s history, as iatrogenic and transient iron overload do not share the poor prognosis of hereditary causes of iron overload of the fetal liver [16, 25]. Furthermore, the same can be said for causes of hepatosplenomegaly other than IUTs [30]. Importantly, standard-of-reference sequences proved sensitive enough to detect iron overload in utero and in vivo without extending the fetal MR protocol.

There are some limitations to our study we want to address. Our study had a rather small sample size, however, indications for IUT are rare, and, in addition, fetal blood transfusions represent a highly sophisticated, not widely available medical procedure. Still, we think that a larger sample size would underline the observed effects as our findings were of robust statistical significance and in themselves coherent. Two fetuses suffered from parvovirus B19 infection and though uncommon, this infection on its own can cause hepato- and splenomegaly in adults and unborn children [32, 33], or hepatitis ranging in severity up to acute liver failure [34] with consequential SI changes. Nevertheless, parvovirus B19 infection may mandate the need for IUT, which limits the exclusion of such cases. Splenomegaly can be present in HDFN prior to IUT and is indicative of severe anemia [35], but this would not explain the hepatomegaly we found in the IUT cohort. And, although hepatic iron overload can already be present before IUT in HDFN due to excessive hemolysis [11], it would not explain the dynamic changes in MRI SI we observed. Post-partum serum ferritin levels were available for only one fetus of the IUT cohort; thus, this data was not included, but ferritin levels were highly elevated. Volumetry of fetal organs in intrauterine MRI poses a difficult task due to the small organ size and potential movement artifacts. To address this and to ensure the quality of our data, two observers conducted the assessment of SI and organ volumetry independently. ICCs showed an excellent level of agreement between the two observers. Finally, this study focused only on the fetal liver and spleen. The effects of transfusion-associated iron overload on the morphology of other organs are challenging to uncover by fetal MRI and, therefore, could not be further considered within the framework of this study.

This study provides initial insight into morphological effects on the developing liver and the spleen following blood transfusions. We were able to demonstrate specific changes in the liver and the spleen following IUT on fetal MRI that may not be apparent on uterine ultrasound. The presented findings represent potential pitfalls when reporting on prenatal MRI and may assist radiologists to better interpret imaging data acquired after IUT.

## Supplementary information

ELECTRONIC SUPPLEMENTARY MATERIAL

## Data Availability

As the data for this manuscript contains sensitive patient information it is not publicly available. Data generated or analyzed during the study is potentially available from the corresponding author after reasonable request and in anonymized form.

## Abbreviations

HDFN: Hemolytic disease of the fetus or newborn
ICC: Intraclass correlation coefficient
IUTs: Intrauterine transfusions
SI: Signal intensity
