# Supplementary material for: Intrauterine blood transfusion causes dose- and time-dependent signal alterations in the liver and the spleen on fetal magnetic resonance imaging
Source: Eur Radiol. 2024 Dec 11;35(3):1605–14. doi: 10.1007/s00330-024-11228-y (PMC11836162; doi:10.1007/s00330-024-11228-y)
Supplement: Supplementary file 1 — ELECTRONIC SUPPLEMENTARY MATERIAL [file 330_2024_11228_MOESM1_ESM.pdf]

Intrauterine blood transfusion causes dose- and time-dependent signal alterations in the liver and the spleen on fetal magnetic resonance imaging

ELECTRONIC SUPPLEMENTARY MATERIAL

Eur Radiol (2024) Schwarz M, Schmidbauer VU, Malik J, et al.

sequence	plane	FOV (mm)	voxel (mm)	slice (mm)	matrix	TR (ms)	TE (ms)	time (min)
T2 SS-GRE	ax/cor/sag	254 x 254 x 84	1.35 x 1.19	4.00	188 x 214 x 41	3.70	1.87	00:34
T2 SS-TSE	ax/cor	230 x 230 x 154	0.90 x 1.37	4.00	256 x 168 x 35	24691	100	00:49
T1 FFE	cor/sag	327 x 327 x 82	1.57 x 1.95	5.00	208 x 167 x 15	131	4.60	00:15
T1 SS-FFE 3D***	cor/sag	450 x 450 x 60	2.08 x 2.08	3.00	216 x 216 x 40	3.50	1.48	00:25
EPI-SWI 3D	cor	300 x 300 x 120	1.97 x 2.01	3.00	152 x 149 x 40	1000	75	00:16
DWIBS	cor	375 x 300 x 125	3.02 x 2.97	5.00	124 x 101 x 25	1848	59	01:32

Suppl. Table S1: Fetal body MRI protocol. DWIBS = diffusion-weighted whole-body imaging with background body signal suppression, EPI = echo planar imaging, FFE = fast field echo, FOV = field of view, GRE = gradient echo, SS = single shot, SWI = susceptibility-weighted imaging, TE = echo time, TR = repetition time, TSE = turbo spin echo.

patie	mat.	gest.	indicat	IU	mL/ses	fetal		fetal	
						befor e	after	before	after
1	29		HDFN	7					
		20+1		#1	30	1.2	12.3	4.0%	40.0%
		21+0		#2	20	8.0	14.4	26.6%	NA
		22+4		#3	30	6.1	12.6	20.0%	NA
		24+4		#4	12	9.2	11.3	29.5%	NA
		27+4		#5	65	5.3	12.0	17.4%	NA
		30+5		#6	80	7.6	12.5	23.4%	NA
		34+0		#7	95	5.4	13.3	13.7%	40.6%
2	35	21+2	Parvov irus	1	50	2.8	14.9	7.5%	41.9%
3	36		HDFN	4					
		24+1		#1	40	5.0	15.3	17.8%	45.4%
		25+6		#2	NA	7.7	NA	24.1%	47.1%
		27+6		#3	40	8.9	NA	28.7%	NA

		30+6		#4	NA	8.3	NA	25.8%	NA
4	33		HDFN	5					
		23+5		#1	60	4.0	14.0	13.4%	46.2%
		25+5		#2	50	8.2	NA	26.7%	NA
		28+6		#3	45	7.9	12.5	26.3%	41.0%
		31+6		#4	95	6.6	13.4	21.0%	42.1%
		33+6		#5	70	8.7	11.8	28.0%	NA
5	31		HDFN	2					
		26+1		#1	40	5.0	11.4	16.0%	29.5%
		29+4		#2	50	2.6	8.3	8.1%	NA
6	18	22+0	TTTS	1	NA	2.6	NA	15.9%	NA
7	35	27+6	HDFN	1	60	3.9	10.0	12.3%	32.5%
8	29		Parvovirus	4					
		19+6		#1	25	6,3	NA	19.8%	NA
		29+2		#2	45	2,5	8,1	8.1%	NA
		29+6		#3	60	5,5	9.8	16.9%	30.6%
		30+6		#4	60	6.7	10.9	21.1%	33.3%

Suppl. Table S2: Details on intrauterine transfusions per patient and per session. Data regarding IUT sessions are provided here. Individual sessions are marked with '#,' total sessions without any prefix. Gestational age is calculated for the time of the IUT. Listed here are the volume transfused per session in mL, fetal blood hemoglobin concentrations in g/dL, as well as fetal hematocrit in percent (%) before and after transfusion.

pa	mat.	deliver	gest.	IU	fetal outcome	maternal
1	29	C-section	34+5	7	Jaundice (bilirubin 10.2mg/dL), phototherapy, discharged two weeks later. Alive, 3 years old (y/o).	Unremarkable
2	35	spont.	25+6	1	Discharged after three weeks, bronchopulmonary dysplasia, abdominal ultrasound at three months unremarkable. Alive, 5 months old.	Unremarkable
3	36	C-section	31+6	4	Prolonged jaundice (bilirubin at 1.5 months 6.1 mg/dL), transfer from neonatal ICU at 1.5 months, hemolysis and transfusion (1x) at 2 months. Alive, 6 y/o.	Unremarkable
4	33	C-section	36+0	5	Postpartum intravenous immunoglobulins, jaundice (week 1 bilirubin 17.2mg/dL), hepatosplenomegaly, steatosis on ultrasound	Unremarkable

					week 2, no cholestasis, postpartum hemolysis at week 2.5, two times transfusion (hematocrit from 24% to 39%), discharge at week 3. Alive, 4 y/o.	
5	31	C-section	30+3	2	Hernia inguinalis requiring surgery, heart insufficiency and cardiomegaly. Alive, 8 y/o.	Intrauterine fetal demise in next pregnancy. Otherwise, unremarkable.
6	18	C-section	37+4	1	Intrauterine hypoxia, mental handicap, microcephaly, structural epilepsy. Alive, 9 y/o. Twin with intrauterine fetal demise.	Next pregnancy again with twins. Otherwise, unremarkable.
7	35	C-section	30+1	1	Discharge from neonatal ICU and transfer to other center after first two weeks. Abdominal ultrasound at month 1 unremarkable, no cholestasis. Alive, 2 y/o.	Unremarkable.

8	29	C-section	31+3	4	Discharge from neonatal ICU and transfer to another center within first week. Alive, 3 y/o.	Unremarkable.

Suppl. Table S3: maternal and fetal outcomes after intrauterine transfusions. Most IUT patients had delivery by C-section, while one spontaneous birth occurred. Gestational age is calculated here for the time of delivery. Several patients were originally from other hospitals; no deaths were reported during follow-up based on the data available at our center. Three patients had prolonged postpartum jaundice, and one required phototherapy, but, in general, the condition was self-limiting. Ultrasounds performed approximately one month after birth found no lasting liver damage, except in one patient (#4) with steatosis and hepatosplenomegaly. Newborns with prolonged postpartum jaundice had four to seven IUT sessions in total per fetus.

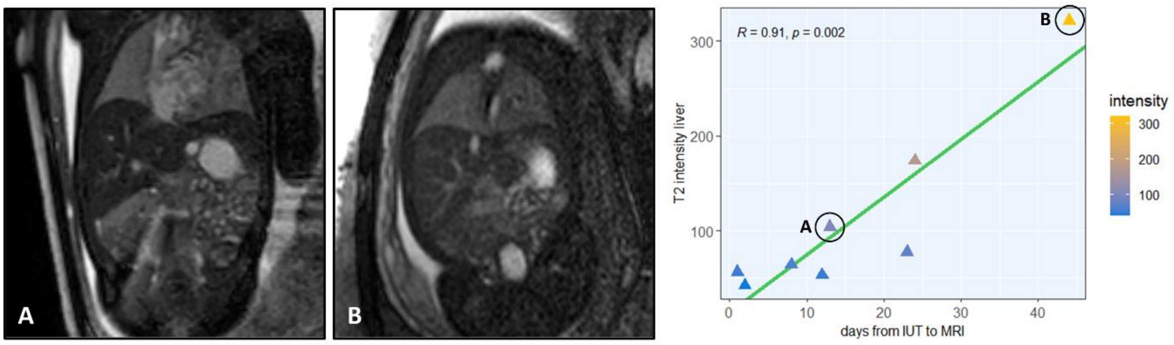

Suppl. Figure S1: Example of time-dependent changes of T2-weighted imaging signal intensity of fetal livers. Panel A shows a fetus with HDFN and gestational age 25+6 who received an IUT 13 days prior to the MRI. A generalized darkening of the liver parenchyma can be observed. Panel B shows a fetus with HDFN and gestational age 28+6 who received an IUT 44 days prior to MRI. Signal intensity of the liver appears normal. For reasons of comparability, images of fetuses with even less days from MRI (left of “A” on x-axis) were not chosen due to differences in gestational age or infection with parvovirus.

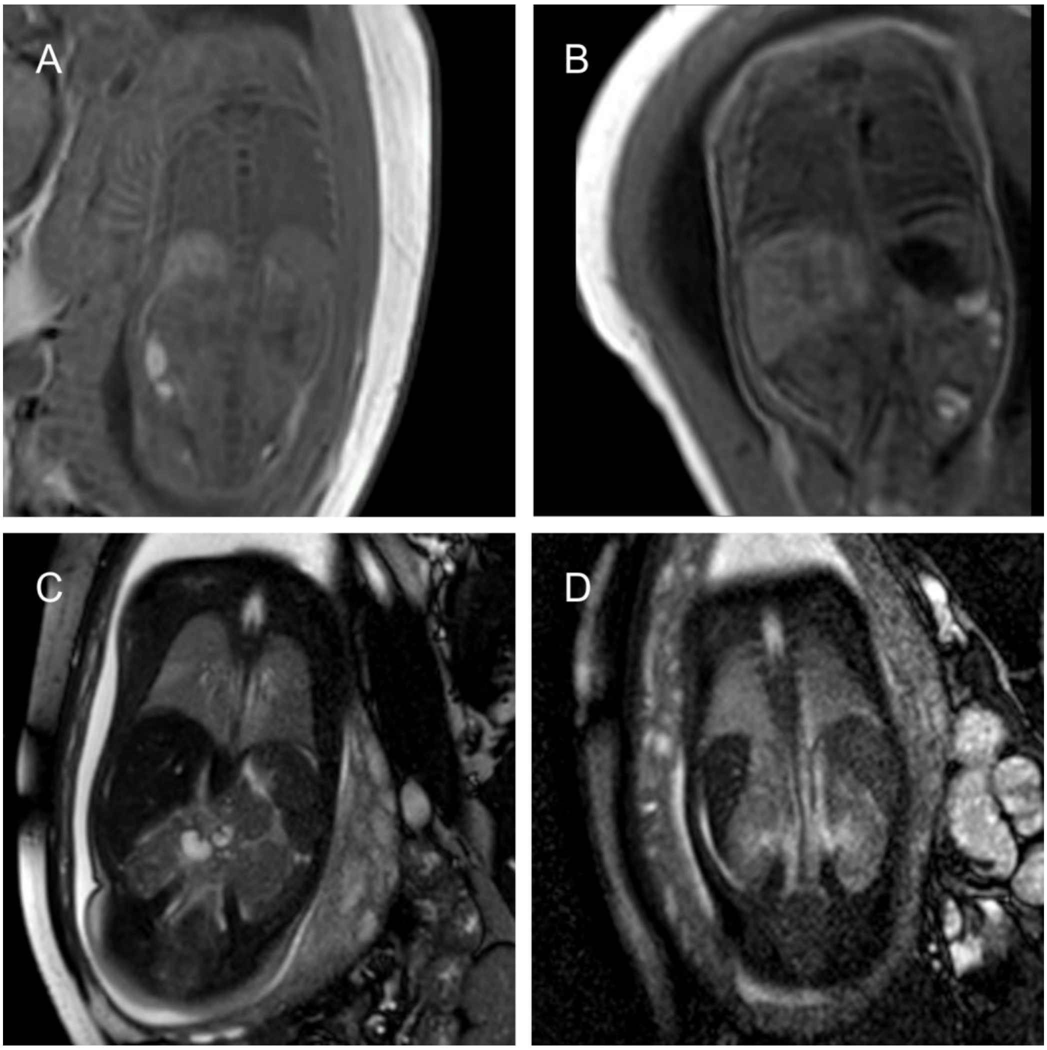

Suppl. Figure S2: Comparison of spleen signal intensity in fetuses with and without IUT on T1- and T2-weighted MRI sequences

A) Picture A shows a T1-weighted sequence of a fetus at a gestational age of 28+4 weeks, who did not receive IUT. Compared to adjacent tissue and organs, the parenchyma of the spleen is slightly hyperintense (SI = 343).

B) T1-weighted sequence of a different fetus at a gestational age of 29+4 weeks, who received only one IUT, but 12 days before MRI acquisition. Similar to fetal livers, a reduction in T1 signal intensity of the spleen parenchyma (SI = 286) can be seen, although not as pronounced.

C) T2-weighted sequence of a third fetus at a gestational age of 31+1 weeks, who received a total of four IUTs, the last one two days prior to MRI acquisition. Similar to the effects we observed in fetal livers, the spleen exhibits a distinct reduction in signal intensity on T2-weighted imaging following IUT (SI = 72), which was most pronounced early after the procedure.

D) For comparison and as an example of the time-dependent effect we observed, a T2-weighted sequence of a different fetus at a gestational age of 28+5 weeks is shown. This fetus received one IUT 24 days prior to MRI acquisition. We found that the signal intensity reduction seen in the first days following the IUT steadily recovered over time. While the fetus in C, who had a transfusion three days before the MRI, had a pronounced reduction in spleen signal intensity (SI = 72), the spleen of this fetus, whose transfusion was more than three weeks prior, was no longer as hypointense (SI = 207).
